# Immunization with an SIV-based IDLV Expressing HIV-1 *Env* 1086 Clade C Elicits Durable Humoral and Cellular Responses in Rhesus Macaques

**DOI:** 10.1038/mt.2016.123

**Published:** 2016-07-26

**Authors:** Donatella Negri, Maria Blasi, Celia LaBranche, Robert Parks, Harikrishnan Balachandran, Michelle Lifton, Xiaoying Shen, Thomas Denny, Guido Ferrari, Maria Fenicia Vescio, Hanne Andersen, David C Montefiori, Georgia D Tomaras, Hua-Xin Liao, Sampa Santra, Barton F Haynes, Mary E Klotman, Andrea Cara

**Affiliations:** 1 Department of Medicine, Duke University Medical Center, Durham, North Carolina, USA; 2 Department of Infectious Diseases, Istituto Superiore di Sanità, Rome, Italy; 3 Department of Surgery, Duke University Medical Center, Durham, North Carolina, USA; 4 Duke Human Vaccine Institute, Duke University Medical Center, Durham, North Carolina, USA; 5 Beth Israel Deaconess Medical Center, Boston, Massachusetts, USA; 6 BIOQUAL Inc., Rockville, Maryland, USA; 7 Department of Therapeutic Research and Medicines Evaluation, Istituto Superiore di Sanità, Rome, Italy

## Abstract

The design of an effective HIV-1 vaccine remains a major challenge. Several vaccine strategies based on viral vectors have been evaluated in preclinical and clinical trials, with largely disappointing results. Integrase defective lentiviral vectors (IDLV) represent a promising vaccine candidate given their ability to induce durable and protective immune responses in mice after a single immunization. Here, we evaluated the immunogenicity of a SIV-based IDLV in nonhuman primates. Six rhesus monkeys were primed intramuscularly with IDLV-Env and boosted with the same vector after 1 year. A single immunization with IDLV-Env induced broad humoral and cellular immune responses that waned over time but were still detectable at 1 year postprime. The boost with IDLV-Env performed at 1 year from the prime induced a remarkable increase in both antibodies and T-cell responses. Antibody binding specificity showed a predominant cross-clade gp120-directed response. Monkeys' sera efficiently blocked anti-V2 and anti-CD4 binding site antibodies, neutralized the tier 1 MW965.26 pseudovirus and mediated antibody-dependent cellular cytotoxicity (ADCC). Durable polyfunctional Env-specific T-cell responses were also elicited. Our study demonstrates that an IDLV-Env-based vaccine induces functional, comprehensive, and durable immune responses in Rhesus macaques. These results support further evaluation of IDLV as a new HIV-1 vaccine delivery platform.

## Introduction

Despite major improvements in the morbidity and mortality associated with HIV-1 infection from introduction of antiretroviral therapy, the development of an effective HIV-1 vaccine for prevention remains a global priority. Recent studies have provided insight into critical elements of an effective vaccine.^1^ The correlates of protection of the only AIDS vaccine trial in humans showing some efficacy, the ALVAC-prime AIDSVAX-boost RV144 trial,^2^ included a direct correlation with IgG anti-V1/V2 non-neutralizing antibodies (Abs), antibody-dependent cellular cytotoxicity (ADCC) and an inverse correlation with anti-Env IgA response in plasma.^3,4,5^ However, the protection was short lived with V1/V2 antibody (Ab) levels waning over 6 months after a boost.^4,6^ Different strategies have been investigated for enhancing the durability of Ab responses against HIV, including repeated boosts, use of novel adjuvants, and a combination of heterologous prime-boost regimens.^7^ Interestingly, a recent study comparing DNA or protein vaccination alone to DNA plus protein boost at the same site in nonhuman primates (NHP) demonstrated that strategies to enhance magnitude of Ab responses differ from those designed to enhance durability. Simultaneous administration of DNA and protein improved magnitude, durability, and increased mucosal dissemination of the induced Abs in immunized rhesus macaques compared to the use of DNA or protein used alone during the immunization.^8^

We recently described a novel delivery system based on self-inactivating (SIN) integrase defective lentiviral vector (IDLV) capable of inducing comprehensive and persistent immune responses after a single immunization in mice.^9,10,11^ IDLVs are nonreplicating and nonintegrating lentivirus-based vectors (LV), which do not express any viral open reading frame (ORF) of parental origin.^12^ Self-inactivation is obtained by deletion of wild-type LTR U3 promoter sequences. Their integration-defective phenotype is achieved by incorporating a mutated form of the integrase (IN) protein in the recombinant LV.^12^ Absence of integrase-mediated integration has been demonstrated both in *in vitro* cell culture systems and *in vivo* in several murine models.^13^ IDLV retains high transduction efficiency and broad tropism associated with conventional LV while the expression of the encoded antigen is driven by circular (episomal) forms of the vector genomes (E-DNA),^14,15^ thus avoiding the potential problems associated with conventional LV integration into genomic DNA. Only the transgene of interest is expressed from E-DNA in the absence of any other parental viral product. Furthermore, because of the intrinsic ability of lentiviruses to infect and express antigens in both dividing and nondividing cells, IDLV can efficiently transduce antigen presenting cells (APC), such as dendritic cells (DC) and macrophages, thus triggering the expansion of antigen-specific T cells.^14,16,17^ Conversely, in cycling cells including T and B cells, IDLV episomes are rapidly diluted as a consequence of cell division.^14^ This important feature increases the safety profile of IDLV compared to the parental integrating counterpart.

HIV- and SIV-based IDLVs have been evaluated as immunization strategies in murine models of infectious diseases inducing strong and protective antigen-specific systemic and mucosal immune responses.^18^ Mice immunized intranasally with IDLV expressing the influenza virus nucleoprotein (Flu-NP) were protected against Influenza virus challenge^19^ and therapeutic vaccination with IDLV expressing the human papillomavirus 16 E7 protein (HPV-E7) as a tumor antigen resulted in the eradication of TC-1-derived tumor in tumor-bearing mice.^20^ Antigen presentation persists for at least 30 days following IDLV immunization,^21^ suggesting that IDLV provides prolonged expression of the encoded antigen, a desirable feature for any vaccine to achieve sustained protection over time. While the efficacy of IDLV immunization at inducing durable and protective antigen-specific immune responses in mice is well established, this vaccine platform has not been evaluated in NHP. We generated a SIV-based IDLV expressing the clade C transmitted founder (T/F) HIV-1 EnvC.1086 gp140 (IDLV-Env) to evaluate safety and immunogenicity in NHP. The SIV-based IDLV was chosen over the HIV-based IDLV because of its higher transduction and antigen expression efficiency in simian DCs.^17^ The HIV-EnvC.1086 gp140 glycoprotein has proven antigenicity and immunogenicity and binds antibodies directed against important neutralizing epitopes.^22,23^ Furthermore, since an effective HIV vaccine needs to protect against T/F virus variants, the use of T/F envelopes as immunogens might elicit more effective immune responses against T/F HIV-1 strains.

In this pilot study, we show for the first time that a single immunization with IDLV-Env in rhesus macaques induced prolonged antibody and T-cell responses, still detectable after 1 year. These responses were boosted following a second IDLV-Env immunization 1 year postprime. The functionality of the induced antibodies and the quality of the T-cell responses support the further development of IDLV as a vaccine delivery platform.

## Results

### Sustained Env-specific Ab responses after IDLV-Env immunization

**Figure 1:**
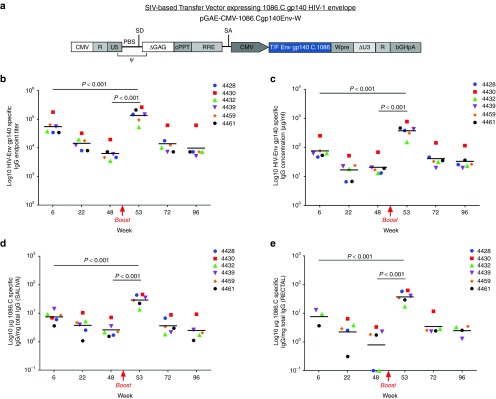
**IDLV-Env immunization induced durable Env-specific antibody responses.** (**a**) Schematic representation of the self-inactivating vector (SIN) used in this study. CMV, cytomegalovirus immediate-early promoter; R, repeat element; U5, 5' untranslated region; U3, 3' untranslated region; PBS, primer binding site; SD, splice donor site; Ψ, packaging signal; cPPT, central polypurine tract; RRE, Rev response element; SA, splice acceptor site; ▵U3, SIN deletion in U3 region of 3' LTR; WPRE, woodchuck hepatitis virus post-transcriptional regulatory element; bGHpA, bovine growth hormone polyadenylation signal. (**b–e**) Anti C.1086 gp140 binding Abs induced by IDLV-Env immunization. The magnitude and durability of anti-Env IgG were measured in six animals in both plasma (**b** and **c**, Ab titer and concentration are shown, respectively) and mucosal secretions (**d** and **e**). Results for saliva and rectal Abs expressed as µg of HIV-specific IgG versus mg of total IgG, in order to account for differences in volume of fluid absorbed by the weck-cel sponges between animals. Lines indicate the predicted mean values at each time point as estimated by a system of piecewise linear regressions including all anti-Env IgG measurements within a structural equation modelling (SEM) frame work.

Monkeys were immunized intramuscularly with IDLV expressing HIV-EnvC.1086 gp140 (IDLV-Env). Presence of Env-specific Abs was assessed over time using plasma samples collected monthly after immunization. All monkeys showed high levels of Env-gp140-specific Abs (**Figure 1b**,**c**). Humoral responses were measured both as endpoint titer (**Figure 1b**) and µg/ml equivalent of rhesus anti-Env IgG B12R1 (**Figure 1c**). After the initial peak at 6 weeks postimmunization (5.6 × 10^4^ and 76.5 µg/ml plasma endpoint titer and concentration, respectively), the levels of Abs slowly decreased overtime. There was a 3.8 times decline in the magnitude of Ab titers between peak and week 22 and a 2.3 times decline between weeks 22 and 48. Overall, the magnitude declined less than one log over a 1-year period, indicating that a single IDLV-Env immunization induced a durable anti-gp140 systemic response.

One year after the first immunization, all monkeys were boosted with the same vector pseudotyped with a different VSV-G serotype (VSV-G_NJ_) to avoid the induction of anti-VSV-G neutralizing Ab directed against the VSV-G_IND_ used in the first immunization. As shown in **Figure 1b**,**c**, the level of anti-C.1086 Env Abs was significantly boosted (*P* < 0.001) in all monkeys with a peak response at 2 weeks postboost (53 weeks postprime: 1.4 × 10^5^ and 380 µg/ml plasma endpoint titer and concentration, respectively). Anti-Env-specific Ab responses were significantly higher than those observed at the peak after the prime (*P* < 0.001). Humoral responses waned overtime after the initial peak, but were still detectable up to 96 weeks with less than twofold decline from week 72 to week 96.

Saliva and rectal swabs were collected monthly to evaluate the presence of Env-specific Abs at mucosal sites. While no anti-Env-specific IgA were detected (data not shown), all monkeys showed anti-Env IgG Abs both in saliva and rectal samples (**Figure 1d**,**e**, respectively). The kinetics of mucosal anti-Env IgG mimicked those observed in plasma. Antibody responses peaked at week 6, decreased overtime but were still present at week 48 postprime. Mucosal anti-Env IgG Abs were significantly boosted after the second immunization (*P* < 0.001) and persisted out to 96 weeks.

### Binding specificity of Ab responses

To determine the epitope specificity of IDLV-Env-induced Abs, linear epitope mapping peptide microarray covering consensus Env (gp160) and vaccine/lab strains (gp120) was performed with serum samples from 6 weeks postprime, 2 weeks postboost, and 13 weeks postboost time-points. Overall, there was a dominant and sustained gp120-directed cross-clade response directed predominantly at V3 in all sera, as shown in **Table 1**, where the highest binding intensity to a single peptide within the epitope is illustrated for each monkey. Conversely, anti-gp41 immunodominant responses were lower and more variable among the animals. All animals showed an increase in total linear gp160 binding at 2 weeks postboost, which decreased by 13 weeks postboost, but was maintained at a level and breadth similar to that observed at 6 weeks postprime.

Two weeks postboost, anti-V3 remained the dominant response, with additional specificities detected in most animals, involving C1, V2, C2, C4, C5, and the gp41 immunodominant region. Moderate changes were observed in the overall clade distribution of gp120 binding responses with binding to gp120 highest against clade C while there was no clear clade-dominance for gp41 binding (**Supplementary Figure S1**).

### IDLV-Env immunization induced functional anti-Env Abs

**Figure 2:**
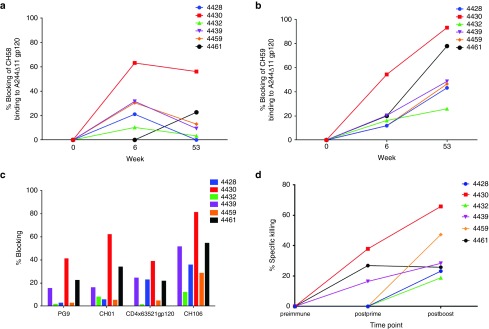
**Conformational Binding Specificities and ADCC activity of IDLV-Env induced Abs.** Blocking of two V2-specific mAbs, CH58 (**a**) and CH59 (**b**), was assessed on plasma samples collected at the peak response of binding Abs, post-prime and post boost. (**c**) Blocking activity against the V1/V2 specific broadly neutralizing mAb (bNab) CH01, the V1/V2-specific bNab PG9 and the CD4-binding site-specific bNab CH106 was assessed on plasma samples postboost. Blocking of soluble CD4 binding to gp120 is also shown. (**d**) Plasma ADCC peak activity. ADCC activity against target cells infected with a molecular clone designed to encode the HIV-1 subtype C 1086.C was measured using the GTL assay. ADCC activity was detected in three out of six animals after the first immunization and in all animals postboost (serum dilution 1:100; cut off: 15%).

A competition assay with monoclonal Abs (mAbs) of known specificities was performed to look for the presence of Abs with similar specificities in the plasma of IDLV-vaccinated animals. Blocking of two V2-specific mAbs, CH58 and CH59 isolated from RV144 vaccinees,^24^ as well as of V1/V2 specific broadly neutralizing mAb (bNAb) CH01^25^ and gp41 specific bNAb 2F5 (ref. 26) was assessed on plasma samples collected at different time points postimmunization. Four of six animals showed blocking of CH58 (**Figure 2a**) and/or CH59 mAbs (**Figure 2b**) postprime and all monkeys had plasma Abs blocking CH59 mAb postboost (**Figure 2b**), while there was no blocking activity of CH01 or 2F5 mAbs (data not shown). Additionally, blocking activity against CH01 bNAb and the V1/V2-specific bNab PG9 (ref. 27) was detected postboost in plasma samples from Mk 4430 and monkey 4461 (**Figure 2c**). Five of six animals showed blocking activity against the CD4-binding site specific bNAb CH106 (ref. 24) and plasma from three animals blocked the binding of soluble CD4 to gp120, postboost (**Figure 2c**). In monkey 4430, the animal with the strongest humoral responses, CH59 and CH106 blocking persisted up to 30 weeks postboost (data not shown).

We then tested for the presence of vaccine-elicited Ab responses against C.1086-infected target cells (**Figure 2d**). Three of six monkeys showed ADCC activity after the prime, while the boost induced Abs able to mediate ADCC in all monkeys. Overall, the ADCC activity was not sustained over time in individual animals and persisted in one of the six animals up to 22 weeks postprime and in one animal up to 13 weeks postboost (latest time point tested) (data not shown).

All animals developed NAbs against the clade C tier 1 isolate MW965.26 (**Table 2**). Interestingly, the peak of NAbs was observed at 14 weeks after a single immunization with IDLV-Env, 8 weeks later than the peak of binding Abs. Neutralizing antibody titers waned after the prime but were detectable continuously throughout the study and were significantly boosted (*P* = 0.002 between weeks 14 and 53) by the subsequent IDLV inoculation (**Table 2**). No NAbs against tier 2 viruses were detected at any of the tested time points.

### IDLV-Env immunization induced sustained T-cell responses in all animals

**Figure 3:**
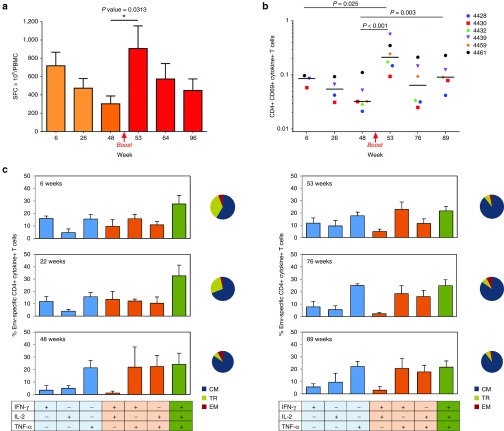
**Env-specific T-cell responses**. (**a**) IFN-γ ELISPOT responses. Persistent cellular immunity was developed in all animals. Results are expressed as mean IFN-γ secreting cells, measured as spot forming cells (SFC) per million PBMC, at different time points. Error bars denote mean with SEM. (**b**) Total Env-specific CD4+ T cells producing IFN-γ, IL-2, and TNFα. IDLV-Env immunization induced Env-specific CD4+ T-cell response in all animals. Data are shown as percent of total CD4+ CD69+ cytokine+ T cells. Dots represent individual responses measured at the indicated time points. Horizontal lines show the median values at a given time point. **(c)** Polyfunctional Env-specific CD4+ T cells at indicated time point postprime (left panels) and postboost (right panels). Percentage of single, double, and triple cytokine producing CD4+ T cells are shown. Phenotypes of CD4+ TFNα+ T cells are illustrated in the pie chart, as a proportion of CM (central memory), TM (transitional memory), and EM (effector memory) T cells.

All monkeys developed specific HIV-C.1086 Env T-cell responses as measured by IFN-γ ELISpot starting from 2 weeks postprime. At peak, the magnitudes of antigen-specific IFN-γ ELISpot responses varied from 420 to 1,420 spot forming cells (SFC)/10^6^ peripheral blood mononuclear cell (PBMC) (**Figure 3a**).The ELISpot responses contracted over time; however, the Env-specific response was still present 48 weeks postprime. All animals showed a significant increase of Env-specific IFN-γ producing cells after the boost (range 335–2,080 SFC). SIV Gag-specific T-cell responses were also induced albeit at lower level than the Env-specific responses and were mainly detectable at early time points postimmunization (data not shown). Since SIV-Gag is present in the vector particles but not encoded in the transfer vector, the low level of Gag responses and the persistence of Env-specific responses over time are consistent with transgene expression in the IDLV-immunized animals.

**Figure 4:**
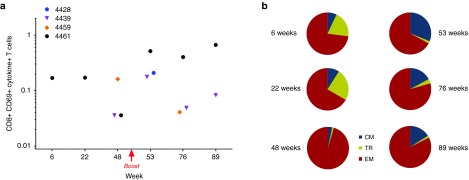
**Env-specific CD8+ T-cell response**. (**a**) IDLV-Env immunization induced Env-specific CD8+ T-cell responses. Data are shown as percent of total CD8+ CD69+ cytokine+ T cells. (**b**) Polyfunctional Env-specific CD8+ T cells from monkey 4461 postprime (left panels) and postboost (right panels). Phenotype of CD8+ TNF-α+ + T cells are shown in the pie chart. CM, central memory; EM, effector memory; TM, transitional memory.

In order to assess the phenotype and quality of vaccine-induced T-cell responses, intracellular cytokine staining (ICS) was performed using Peripheral blood lymphocytes (PBL) collected at different time points postprime and postboost (**Figure 3b**). The majority of the vaccine-elicited Env-specific T cells were CD4+ cells secreting IFN-γ, IL2, and TNF-α, most of them with a central memory (CM) phenotype (CD28+/CD197+ cells). The CM cells increased overtime and represented about 90% of the total Env-specific CD4+ T cells after the boost (**Figure 3c**). Induction of specific CD8+ T cells producing IFN-γ and TNF-α was detected in three of six monkeys, but clearly evident and persistent only in monkey 4461 at both postprime and postboost time points (**Figure 4a**). Of note, the majority of these cells were effector memory (EM) cells (CD28/CD197 negative) (**Figure 4b**). The percentage of EM CD8+ T cells was higher after the prime and slightly decreased after the boost and remained the dominant phenotype.

### Absence of replication competent virus in the plasma of IDLV-immunized monkeys

Although unlikely, it is possible that replication-competent SIV is generated *in vivo* following IDLV immunization. To confirm the absence of replicating SIV *in vivo*, plasma samples from monkeys immunized with IDLV-Env and from naive monkeys (negative control) were tested for the presence of SIV RNA. To increase the sensitivity, amplification was performed using RNA extracted from 7.5 ml of plasma from each animal collected at week 50 postboost (21 copies/ml sensitivity, using 3 ml of plasma per run). No detectable SIV sequences were found after two immunizations with IDLV-Env, consistent with the absence of circulating SIV (data not shown).

## Discussion

The development of a protective vaccine against HIV remains a major challenge. In the present work, we conducted a pilot study in NHP to evaluate for the first time the immune responses induced by a SIV-based IDLV expressing the T/F HIV-1 EnvC.1086 gp140 (IDLV-Env). IDLV-Env immunization efficiently induced functional and durable responses that declined by less than fourfold during the first 6 months after the prime and were measurable up to 1 year from the immunization. In the RV144 trial, which showed a moderate 31.2% protection,^2,4^ the magnitude of binding Abs declined by 10-fold in the same period^29^ suggesting that a vaccine strategy capable of inducing more durable antibody responses like IDLV-Env could increase vaccine efficacy. A boost given 1 year from the prime with the same vector induced a significant increase in both humoral and cellular responses. Durable HIV-Env-specific IgG Abs were also present at mucosal sites, including saliva and rectal secretions, mirroring the trend observed in plasma, while no Env-specific IgA was detected. IgG can mediate active humoral protection in various mucosal locations and the presence of anti HIV envelope IgG in rectal mucosa, following intramuscular immunization, correlated with delay in virus acquisition in a SIV challenge model.^30^ Furthermore, the presence at mucosal surfaces of passively infused NAbs mediated protection against SIV challenge in rhesus macaques,^31^ supporting a protective role for humoral responses at the portal of virus entry.

Immunization with IDLV expressing HIV-gp140-induced Abs directed at different epitopes on the HIV-1 gp120 glycoprotein, with a higher frequency of V3-specific Abs. Lower response was detected versus gp41 linear epitopes. In acute HIV-1 infection, the dominant initial plasma antibody (Ab) response is targeted to gp41 and the majority of gp41-binding antibodies are crossreactive responses of pre-existing B cells originally activated by non-HIV antigens.^32^ Interestingly, a similar diversion of the antibody response toward gp41 might explain the lack of efficacy of a recently reported vaccination strategy with DNA/Ad5 expressing the HIV-1 Env-gp140 (HVTN 505 study). In this trial, the predominant Abs induced were directed against gp41,^33,34^ whereas previous gp120-only vaccines in humans^35^ and rhesus macaques^36^ induced a robust gp120-reactive memory B-cell response. This vaccine was the first vaccine containing the ectodomain of the Env gp41 component, covalently linked to gp120, to be tested in an efficacy trial.^33^ In our study, the immunization with IDLV expressing HIV-gp140-induced Abs mainly directed to the gp120 protein, as assessed by linear epitope mapping. Further studies with gp120 and gp41 proteins would be needed to assess conformational epitopes as well. IDLV-Env vaccination induced functional antibodies targeting multiple epitopes on the HIV-1 envelope as shown by the ability to block mAbs with different specificities, including anti-V2 and anti-CD4bs mAbs, and the ability to mediate ADCC, shown to correlate with a decreased risk of infection in the RV144 vaccine trial.^3,4^

Persistent levels of NAbs against clade C tier 1 MW965.26 were elicited, while no neutralizing activity against the autologous (tier 2) virus was detected using the TZM-bl assay. Similar results were reported in a previous NHP study using the same HIV-1 C.1086 envelope expressed by MVA or DNA.^22^ Although an ideal HIV vaccine should induce bNAbs able to neutralize a broad panel of viral strains, so far no vaccine strategies evaluated in NHP and in humans have successfully induced bNAbs, including the moderately protective RV144 vaccine regimen, which elicited tier 1 but not tier 2 neutralizing responses.^37,38,39^

Env-specific T-cell responses were developed in all animals with good durability, declining by only onefold during the first 6 months postprime and by only twofold at 1 year after immunization. Polyfunctional CD4+ CM T cells producing IFN-γ, TNF-α, and IL-2 cytokines were elicited. The induction of HIV-specific CD4+ T cells may increase the target cell pool for HIV infection and may have implication for HIV replication.^40^ Two human trials (VAX003 and VAX004) testing the recombinant HIV gp120 protein vaccine showed that despite induction of gp120-specific CD4 T cells, no enhancement of HIV infection was observed following vaccination.^41,42^ Moreover, in the RV144 HIV vaccine trial, decreased HIV acquisition was observed with a vaccine that induced HIV-specific CD4 T cells.^2^ Overall, these data suggest that the beneficial protective immunity induced by vaccines including HIV Envelope compensates for the potential negative effect posed by the vaccine-activated CD4 T cells.^7,43^ Understanding the role that vaccine induced antigen-specific CD4+ T cells have in HIV/SIV acquisition risk is fundamental and will be addressed in future SIV challenge studies using IDLV as a delivery system.

Interestingly, in the monkey showing the highest T-cell response (4461), persistent CD8+ EM T cells producing IFN-γ and TNF-α were detected after the prime and postboost. This is consistent with previous murine studies where CD8+ EM T cells were generated and maintained overtime using a different IDLV-Env^11^ and IDLV expressing other antigens.^10,16^ Importantly, HIV-specific EM T cells are required to counteract the SIV infection in NHP model of infection.^44^

The most compelling advantage of IDLV as a delivery system is the durability of humoral and T-cell immune responses induced after a single intramuscular immunization in monkeys. To our knowledge, no other vaccine strategies based on the use of nonreplicating recombinant vectors or soluble antigens showed similar durability of responses 1 year after a single immunization in NHP. Frequent immunizations with either homologous or heterologous boosts are usually required to obtain such responses in monkeys. Adenoviral vector (Ad)-based vaccines, have also been shown to induce potent and durable T-cell responses following a single intramuscular immunization.^45,46^ However, the Ab titers induced with Ad immunization are usually low and require homologous and/or heterologous boost(s) with MVA or protein.^45,46^ In addition, pre-existing anti-vector immunity may decrease the efficacy of vaccination with MVA and Ad-specific CD4+ T cells induced upon immunization with some Ad serotype are more susceptible to HIV infection in NHP and humans.^47^ In a recent report utilizing a replication-competent CMV-based vector as an SIV vaccine in rhesus monkeys, the EM CD8+ T-cell response appeared to be a main contributor to protection and effective containment of SIV following mucosal SIV challenge.^48^ Similarly, live-attenuated SIV-based virus vaccines, although not considered safe, protected monkeys against single or repeated challenge with pathogenic virus strains.^49^ The chronic antigen exposure provided by these vectors may have contributed to the generation of protective responses, suggesting that a strategy that provides prolonged expression of HIV antigens will more effectively elicit durable functional Ab responses and virus-specific EM CD8+ T-cell responses required for preventing or containing HIV-1 replication *in vivo*.

Previous murine studies demonstrated prolonged transcription and antigen expression following intramuscular IDLV vaccination, with expression at the site of injection still present at 3 months from the injection.^50^ It is likely that similar prolonged expression of IDLV delivered antigens in the NHPs was responsible for the maintenance of the functional immune responses over time.

The use of the VSV-G glycoprotein in the pseudotyped IDLV could also contribute to the prolonged vector-induced immune responses. Adherence of VSV-G-pseudotyped LV to transduced cells leads to additional cycles of transduction over time *in vitro* and *in vivo*.^51,52,53^ This could be a potentially important and overlooked advantage of recombinant VSV-G-pseudotyped IDLV. Interestingly, HIV virus-like particles (VLP) pseudotyped with VSV-G showed higher immunogenicity in NHP than VLP that lacked VSV-G.^54^

In the only reported study where HIV-based integrating LV (expressing SIV-Gag) were used to immunize NHP,^55^ there was a good Gag-specific T-cell response with partial protection after challenge similar to other vaccine strategies in monkeys challenged with SIV, including those based on heterologous/multivalent immunization with DNA and rAd.^55,56,57^ We used a SIV-based IDLV since it has a more favorable expression profile compared to HIV-based IDLV in simian primary cells.^17^ This is likely due to both the presence of species-specific restriction factors, as previously shown using HIV-1 in simian cells,^58,59^ and to the presence of SIV-Vpx in the SIV-based IDLV, which was shown to improve transduction of human and simian DC, thus increasing their ability to act as functional APC.^17^

The use of self-inactivating IDLV offers important safety advantages compared to parental integrase competent LV, including the exponentially lower integration rate which minimizes adverse events secondary to integration sites and the lower probability of generating replication-competent lentivirus (RCL). Importantly, RCL events have not been reported for integrating LV using sensitive techniques in up to a total of 1.4 × 10^10^ TU of vector from 10 independent 14-l production lots.^60,61,62^ Vector mobilization via encapsidation of RNA transcripts from superinfection with wild-type virus is also highly unlikely as E-DNA is unstable and lost in dividing T cells.^12,13^ Consistent with these enhanced safety features, no replicating wild-type SIV was detected *in vivo* in the plasma of monkeys immunized twice with IDLV-Env. However, the possibility of generating recombinant virus between IDLV and replicating SIV/SHIV after challenge remains to be determined.

In conclusion, IDLV represents a novel vaccine platform to be further exploited alone or in combination with other vectors or soluble protein/adjuvants strategies to improve the durability of immune responses in the NHP model of AIDS. Further efforts in immunogen design are necessary to generate an efficient HIV vaccine able to induce potent bNAbs. Recent data suggest that the elicitation of anti-HIV-1 broadly neutralizing antibodies will require immunization with a succession of related immunogens.^28,63^ The durability of immune responses induced with IDLV, coupled with such an immunogen strategy to elicit bNabs could result in a sustained protective response. Future challenge studies with IDLV will evaluate the sequential immunogens hypothesis as well as IDLV's safety features to determine whether IDLV-based vaccines could be advanced to human clinical trials.

## Materials and Methods

***Construction of IDLV-Env plasmid.*** The clade C transmitted founder (T/F) HIV-1 Env C.1086 gp140 glycoprotein^23^ was cloned into a SIV-based self-inactivating lentiviral transfer vector^9^ downstream of the internal CMV promoter (pGAE-CMV-C.1086gp140Env-Wpre) (**Figure 1a**). The transfer vector pGAE-CMV-GFP-Wpre expressing the green fluorescent protein (GFP), the IN-defective packaging plasmid pAd-SIV-D64V, containing the D64V aminoacid mutation in the integrase catalytic triad to abolish the IN activity and the Vesicular Stomatitis virus envelope G protein (VSV-G) pseudotyping vectors from Indiana or New Jersey serotypes (pVSV.G_IND_ and pVSV.G_NJ_), have been previously described.^55,64^

***Vector production and validation.*** The human epithelium kidney 293T Lenti-X cells (Clontech Laboratories, Mountain View, CA) were maintained in Dulbecco's Modified Eagles medium (Thermo Fisher Scientific, Waltham, MA) supplemented with 10% fetal bovine serum (GE Healthcare Life Sciences, HyClone Laboratories, South Logan, UT) and 100 units/ml of penicillin–streptomycin–glutamine (PSG) (Thermo Fisher Scientific). For production of recombinant IDLV, 3.5 × 10^6^ Lenti-X cells were seeded on 100 mm diameter Petri dishes and transfected with 12 µg per plate of a plasmid mixture containing transfer vector, packaging plasmid and VSV.G plasmid in a 6:4:2 ratio, using the JetPrime transfection kit (Polyplus Transfection Illkirch, France) following the manufacture's recommendations. At 48 and 72 hours post-transfection, culture supernatants were cleared from cellular debris by low-speed centrifugation and passed through a 0.45 μm pore size filter unit (Millipore, Billerica, MA) . Filtered supernatants were concentrated by ultracentrifugation for 2 hours at 23.000 RPM on a 20% sucrose cushion. Pelleted vector particles were resuspended in 1× phosphate-buffered saline (PBS) and stored at −80 °C until further use. Each IDLV-Env stock was titered using a reverse transcriptase (RT) activity assay (RetroSys RT ELISA kit, Innovagen, Lund, Sweden) and the corresponding transducing units (TU) calculated by comparing the RT activity of each IDLV-Env stock to the RT activity of IDLV-GFP stocks with known infectious titers.^11,64^ HIV-Envelope expression was confirmed on 293T cells transduced with IDLV-Env on both supernatants and cell pellets by gp120 ELISA (HIV-1 gp120 Antigen capture assay; Advanced Bioscience Laboratories, Rockville, MD).

***Animals and immunization protocol.*** The six Indian origin rhesus macaques (Macaca mulatta), used in this study were housed at BIOQUAL, in accordance with the recommendations of the Association for Assessment and Accreditation of Laboratory Animal Care International Standards and with the recommendations in the Guide for the Care and Use of Laboratory Animals of the United States - National Institutes of Health. The Institutional Animal Use and Care Committee of BIOQUAL approved these experiments (study #15–029). When immobilization was necessary, the animals were sedated by intramuscular injection with 10 mg/kg of ketamine HCl. All efforts were made to minimize suffering. Details of animal welfare and steps taken to ameliorate suffering were in accordance with the recommendations of the Weatherall report, “The use of non-human primates in research”. Animals were housed in an air-conditioned facility with an ambient temperature of 21–25 °C, a relative humidity of 40–60% and a 12 hour light/dark cycle. Animals were socially housed when possible or individually housed if no compatible pairing could be found. Animals also received appropriate environmental enrichment. The animals were housed in suspended stainless steel wire-bottomed cages and provided with a commercial primate diet and fresh fruit twice daily, with water freely available at all times. Animals were immunized intramuscularly with IDLV-Env with 3 × 10^8^ TU/animal/immunization in 2-ml injection volume divided into two sites (left and right thighs). Peripheral blood, saliva, and rectal swabs were obtained prior to immunization, 2 weeks after immunization and at monthly intervals throughout the study. Clotted blood from immunized monkeys was centrifuged to collect sera and EDTA anticoagulated blood was centrifuged over Ficoll (Ficoll-Paque) and plasma and PBMC layers were collected in separate tubes. Plasma and serum samples were stored at 80 °C; PBMCs were cryopreserved and stored in liquid nitrogen.

***Antibody recovery from mucosal secretions.*** Mucosal Abs were eluted from the Weck-Cel sponges as described.^22^ Briefly, the elution buffer for a single elution was prepared by adding 6 μl of 100× protease inhibitor cocktail set 1 (Millipore, Billerica, MA) to 594 μl of 1× PBS containing 0.25% bovine serum albumin (Sigma-Aldrich, St. Louis, MO). Before elution, sponges were thawed on ice, transferred to the upper chamber of a filter less Spin-X column (Corning Life Sciences, New York, NY) and 300 μl of ice-cold elution buffer was added directly onto the sponge for 5 minutes. The columns were then centrifuged at 16,000 g for 5 minutes. A second round of elution was performed using a fresh aliquot of elution buffer. The samples were incubated for 10 minutes before centrifugation at 16,000 g for 20 minutes. The eluted fluid was filtered through a Spin-X column containing a 0.22-μm filter (Corning Life Sciences). Samples and buffers were incubated on ice throughout the procedure.

***Antibody characterization by ELISA.*** High binding EIA/RIA 384-well plates (Corning Life Sciences) were coated overnight with 3 µg/ml of either C.1086gp140 protein, goat anti-monkey IgG (Rockland, Gilbertsville, PA), or goat anti-monkey IgA (Rockland, Gilbertsville, PA) in coating buffer (KPL). After one wash with washing buffer (1× PBS + 0.1% Tween 20) plates were treated with blocking buffer/assay diluent (PBS containing 4% [wt/vol] whey protein–15% normal goat serum–0.5% Tween 20–0.05% sodium azide). Serial dilutions of plasma, saliva and rectal secretions were added to the plates in duplicates. Abs were detected by horse radish peroxidase-conjugated, polyclonal goat anti-monkey IgG (Rockland) or IgA (Rockland, Gilbertsville, PA) and by adding the ABTS-2 peroxidase substrate system (KPL, Gaithersburg, MD). *Macaca mulatta* purified IgG and IgA (NIH Nonhuman Primate Reagent Resource, Bethesda, MD) were used to develop standard curves, and the concentration of IgG or IgA antibody was calculated relative to the standard using a 5-parameter fit curve (Softmax, Molecular Devices). Endpoint titers were determined as the reciprocal of the highest dilution giving an absorbance value at least equal to threefold that of background (preimmune biological sample from each monkey). Results for saliva and rectal Abs are expressed as µg of HIV-specific antibodies (IgG/IgA) versus mg of total IgG/IgA, in order to account for differences in volume of fluid absorbed by the weck-cel sponges between animals.

***Antibody blocking assay.*** Ab blocking using ELISA was carried out as described.^65^ ELISA plates (384-well plates; Costar 3700, Corning Life Sciences) were coated with 30 ng/well Env gp120 overnight at 4 °C and blocked with blocking buffer/assay diluent for 1 hour at room temperature. All assay steps were conducted in assay diluent (except the substrate step) and incubated for 1 hour at room temperature followed by washing with PBS–0.1% Tween 20. Samples were diluted and incubated in triplicate wells. Biotinylated-Mab binding was detected with streptavidin-horse radish peroxidase at 1:30,000 (Thermo Fisher Scientific) followed by TMB substrate (KPL, Gaithersburg, MD). For sCD4 blocking, 10 µl of a saturating concentration of soluble CD4 (Progenics Pharmaceuticals, Inc., Tarrytown, NY) was added following sample incubation step. Binding of sCD4 was then detected with biotin-anti-CD4 mouse Mab OKT4 (eBioscience, Inc., San Diego, CA) followed by streptavidin-horse radish peroxidase. Plates were read with a plate reader at 450 nm. Triplicate wells were background subtracted and averaged. Percent blocking was calculated as follows: 100-(sample triplicate mean/no binding control mean) × 100.

***Linear epitope mapping.*** Serum epitope mapping of heterologous strains was performed as described^3,66,67,68^ with minor modifications. Briefly, a peptide library of overlapping peptides (15-mers overlapping by 12), covering 7 full-length HIV-1 gp160 Env consensus sequences (clades A, B, C, and D, group M, CRF1, and CRF2) and 6 vaccine and laboratory strain gp120 sequences (A244_1, TH023_1, MN_B, 1086_C, TV1_C, and ZM651_C), was printed onto epoxy glass slides (JPT Peptide Technologies GmbH, Germany). Microarray binding was performed using the HS4800 Pro Hybridization Station (Tecan, Männedorf, Switzerland). All arrays were blocked with blocking buffer (PBS+1% milk + 5% NGS + 0.05% Tween20) for 1 hour at 30 °C, followed by a 2 hour incubation at 30 °C with sera diluted 1:50 in blocking buffer. Arrays were incubated for 45 minutes at 30 °C with goat anti-Hu IgG conjugated with DyLight649 (Jackson ImmunoResearch, PA) (1.5 µg/ml final concentration) diluted with blocking buffer. Washes between all steps were with PBS containing 0.1% Tween20. Arrays were scanned at a wavelength of 635 nm using an Axon Genepix 4300 Scanner (Molecular Devices, Sunnyvale, CA) at a PMT setting of 580, 100% laser power. Images were analyzed using Genepix Pro 7 software (Molecular Devices, LLC., Sunnyvale, CA). Binding intensity of the post immunization serum to each peptide was corrected with its own background value, which was defined as the median signal intensity of the pre bleed serum for that peptide plus three times the standard errors among the three subarray replicates present on each slide.

***Neutralization assays.*** Neutralization of Env-pseudotyped viruses was measured in 96-well culture plates using Tat-regulated firefly luciferase (Luc) reporter gene expression to quantify reductions in virus infection in TZM-bl cells.^69,70^ Neutralization of Env.IMC.LucR viruses was measured using Tat-regulated Renilla Luc reporter gene expression A3R5 cells.^71^ A panel of five viruses was used to measure serum neutralization: HIV MW965 (tier 1), HIV Ce1086_B2.LucR.T2A.ecto (autologous, tier 2), HIV Q23.17 (Clade A Tier 1), HIV Ce1176_A3.LucR.T2A.ecto (tier 2) and HIVCe0393_C3.LucR.T2A.ecto (Clade C tier 1). Heat-inactivated (56 °C, 1 hour) serum samples were assayed at threefold dilutions starting at 1:20. Neutralization titers (50% inhibitory dose (ID50)) are the serum dilutions at which relative luminescence units (RLU) were reduced by 50% compared to RLU in virus control wells after subtraction of background RLU in cell control wells. A response was considered positive if the post-immunization ID50 was 3 times higher than the preimmune ID50.

***ADCC assay.*** ADCC activity against HIV-1 infected cells was measured using the ADCC-GranToxiLux (GTL) assay as described.^72,73^ Target cells were CEM.NKRCCR5 cells (NIH AIDS Reagent Program, Division of AIDS, NIAID, NIH: CEM.NKR-CCR5 from Dr. Alexandra Trkola) infected with a replication-competent infectious molecular clone encoding the HIV-1 C.1086 isolate (accession number FJ444395) *env* gene in cis within an isogenic backbone that expresses the Renilla luciferase reporter gene and preserves all viral open reading frames.^74,75^ The effector cells were PBMC isolated from a HIV-1 seronegative human donor heterozygous for 158F/V polymorphic variants of Fcγ receptor 3A.The results were calculated as maximal % specific lysis (%SL) after subtracting the activity observed before immunization (after background subtraction). Results were considered positive if the %SL >15%.

***IFN-γ ELISpot assay.*** Multiscreen ninety-six well plates were coated overnight with 100 μl per well of 5 μg/ml anti-human interferon-γ (IFN-γ) (B27; Becton, Dickinson and Company, Franklin Lakes, NJ) in endotoxin-free Dulbecco's-PBS (D-PBS). Plates were washed three times with D-PBS containing 0.1% Tween-20, blocked for 2 hours with Roswell Park Memorial Institute 1640 medium (RPMI) containing 10% fetal bovine serum and incubated with peptide pools and 2 × 10^5^ PBMCs in triplicate in 100 μl reaction volumes. Each peptide pool (1 μg/ml) was comprised of 15 amino acid peptides overlapping by 11 amino acids. The pools covered the entire HIV-1 C.1086 Env. Following 18-hour incubation at 37 °C, plates were washed nine times with D-PBS containing 0.1% Tween-20 and once with distilled water. Plates were then incubated with 2 μg/ml biotinylated rabbit anti-human IFN-γ (U-CyTech biosciences, Utrecht, The Netherlands) for 2 hours at room temperature, washed six times with D-PBS containing 0.1% Tween-20, and incubated for 1.5 hours with a 1:500 dilution of streptavidin-AP (Southern Biotechnology, Birmingham, AL). After five washes with D-PBS containing 0.1% Tween-20 and three washes with D-PBS alone, the plates were developed with bromochloroindolyl phosphate–nitro blue tetra-zolium (BCIP-NBT) chromogen (Thermo Fisher Scientific), stopped by washing with tap water, air dried, and read with an ELISpot reader (Cellular Technology Limited (CTL), Cleveland, OH) using ImmunoSpotAnalyzer software (Cellular Technology Limited (CTL)). Samples were considered positive if number of SFC was above twice that of the background (unstimulated) and > 50 SFC per million PBMC.

***PBL stimulation and intracellular cytokine staining.*** PBMC were isolated from EDTA-anticoagulated blood (GE Healthcare Bio-Sciences, Pittsburgh, PA) and cryopreserved. Cells were later thawed and rested 4 hours at 37 °C in a 5% CO2 environment. PBMC were then incubated for 6 hours in the presence of either RPMI containing 10% fetal bovine serum (unstimulated), PMA/ionomycin as positive control, or pool of HIV-1 C.1086 Env and SIV Gag peptides. All cultures contained a protein transport inhibitor, monensin (Golgi Plug; Becton, Dickinson and Company), and 1 μg/ml of anti-CD49d (Becton, Dickinson and Company). Cultured cells were then stained with a cell viability marker (Fixable Live Dead Yellow, Thermo Fisher Scientific) and pretitered quantities of anti-CD4 APC-H7 (L200; Becton, Dickinson and Company), anti-CD8 BV570 (RPA-T8, BioLegend, San Diego, CA), anti-CD28 PE Cy7 (28.2, eBioscience) and anti-CD197 PerCP-Cy5.5 (150503, Becton, Dickinson and Company). Following fixation and permeabilization with Cytofix/Cytoperm solution (Becton, Dickinson and Company), cells were stained with anti-CD3 V450 (SP34.2, Becton, Dickinson and Company), anti-CD69-ECD (TP1.55.3, Beckman Coulter, Brea, CA), anti-IFN-γ APC (B27, Becton, Dickinson and Company), anti-TNF-α-FITC (MAb11, Becton, Dickinson and Company) and anti-IL-2 PE (MQ1-17H12, Becton, Dickinson and Company). Samples (500,000 events were per sample) were analyzed on a LSR II instrument (Becton, Dickinson and Company, Franklin Lakes, NJ) using FlowJo software (version 9.3.1). Samples in which the percentage of cytokine-staining cells were at least twice that of the background or in which there was a distinct population of cytokine brightly positive cells were considered positive.

**In vivo *RCL determination.*** To assess the remote possibility that replication competent lentivirus (RCL) was generated *in vivo* following IDLV-Env immunization, viral load testing was performed as described,^76,77^ on plasma samples collected from all monkeys at 10 months post-IDLV-Env boost. To increase the test sensitivity, RNA was extracted from a higher volume of plasma (7.5 ml) than standard assay (21 cp/ml sensitivity, using 3 ml of plasma per run).

Viral RNA was extracted from plasma using a Qiagen QIA symphony (Valencia, CA) Virus/Bacteria Midi kit on Qiagen's automated sample preparation platform QIA symphony SP and eluted in 30 μl of elution buffer. All subsequent reactions were setup in duplicates using Qiagen's automated polymerase chain reaction (PCR) setup platform, the QIAgility. Fifteen μL of viral RNA was annealed to a target specific reverse primer 5'- CACTAGGTGTCTCTGCACTATCTGTTTTG -3' and reverse transcribed using Super Script III RT (Invitrogen, Foster City, CA) and PCR nucleotides (Roche, Indianapolis, IN) along with RNAse Out (Invitrogen) using an optimized version of the manufacturer's protocol. cDNA was treated with RNAse H Applied Biosystems (Foster City, CA) according to manufacturer's protocol. Ten μL of each cDNA were then used to setup a real-time PCR using Gene Expression Mastermix (Applied Biosystems) along with target specific labeled probe 5'-/56-FAM/CTTCCTCAGTGTGTTTCACTTTCTCTTCTGCG/3BHQ_1/-3' and forward 5'-GTCTGCGTCATCTGGTGCATTC-3' and reverse primers 5'-CACTAGGTGTCTCTGCACTATCTGTTTTG-3' (custom synthesis by Integrated DNA Technologies). Real-time PCR was performed on an Applied Biosystems Step One Plus platform using the standard curve protocol. The RNA standard was transcribed from the pSP72 vector containing the first 731 bp of the SIVmac239- or SIVsmE660-gag gene using the Megascript T7 kit (AmbionInc, Foster City, CA), quantitated by optical density, and serially diluted to generate a standard curve. The quality of the RNA standard was assessed using an Agilent Bioanalyzer with RNA Nano 6000 chips (Agilent Inc., Santa Clara CA).

***Statistical analysis.*** The temporal trend of antibody response (i.e., plasma/mucosal binding antibodies and neutralizing antibodies) and T cell response was analyzed using a system of piecewise linear equations in order to jointly evaluate the relationships of each outcome at each time point, allowing for correlated errors. The analysis was conducted in STATA13 (StataCorp LP, College Station, TX) within a structural equation modelling (SEM) frame work.^78,79^ The log likelihood estimation procedure was used to fit the model to the data.

**SUPPLEMENTARY MATERIAL** **Figure S1.** Cross clade median epitope binding intensity in the 6 immunized animals.

## Author Contributions

D.N. designed the study, analyzed the data, and wrote the manuscript. M.B. constructed plasmids, produced and tested all the vector preparations used for animal immunization, performed all the ELISAs on plasma and mucosal samples, analyzed the data and wrote the manuscript. C.L. and D.C.M. oversaw the neutralization assays and provided input on study design. R.P. performed the blocking assay. H.B. and M.L. performed ICS and ELISPOT assays. X.S. performed epitope mapping. T.D. oversaw SIV testing. G.F. oversaw ADCC assays. G.D.T. oversaw the direction of the epitope mapping assays, provided input on data analysis and interpretation. X.L. provided reagents. S.S. oversaw the planning and direction of the T cell assays. M.F.V. performed statistical analysis. H.A. participated in study implementation. B.F.H. provided input on study design, planning of experiments and interpretation of the data. M.E.K. and A.C. oversaw the planning and direction of the project including analysis and interpretation of the data and editing of the manuscript.

## Figures and Tables

**Table 1:**
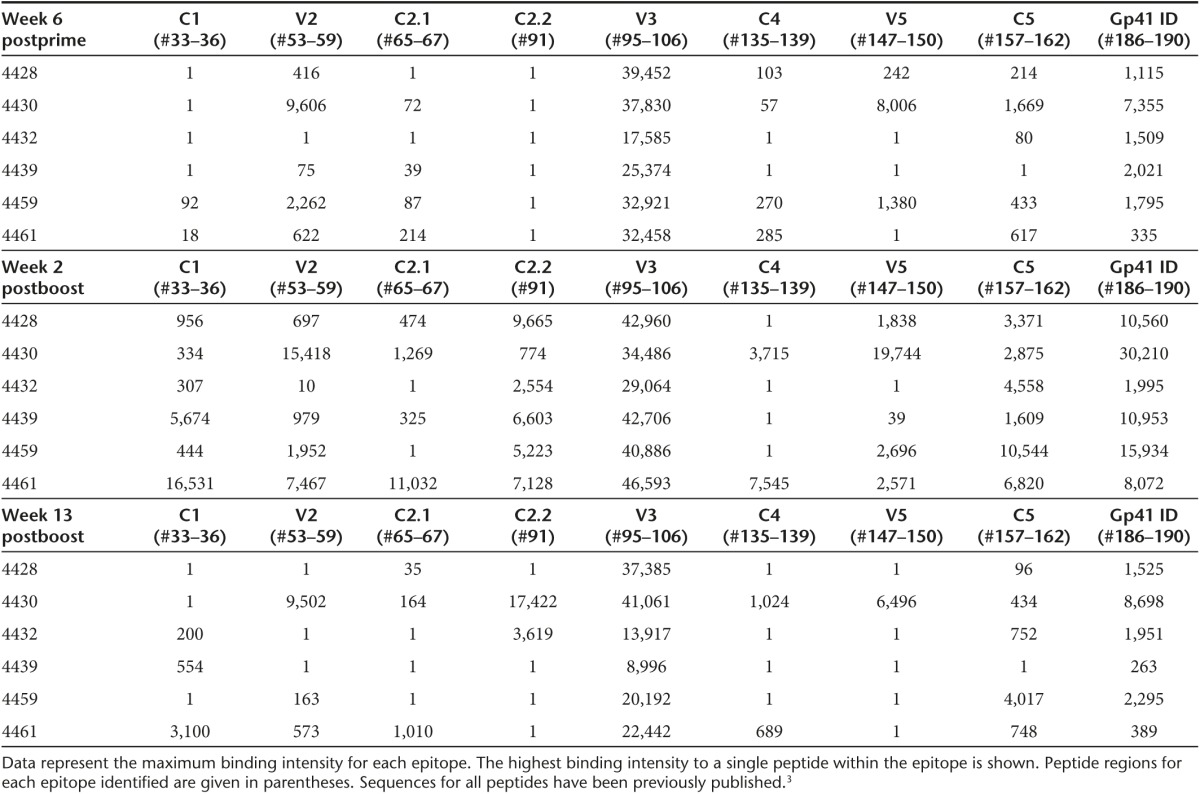
Magnitude of HIV-1 gp160 epitopes binding by plasma Abs

**Table 2:**
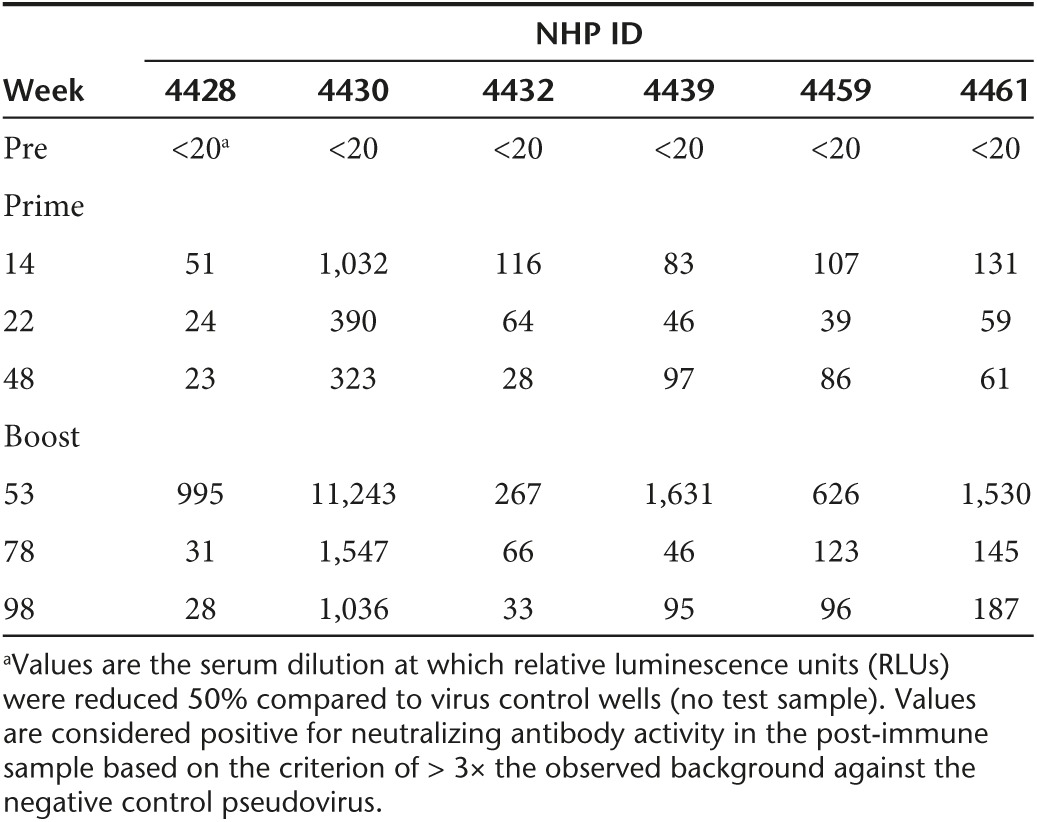
Serum neutralization activity against the clade C Tier 1 virus MW965.26
