# Supplementary material for: Immunization with an SIV-based IDLV Expressing HIV-1 Env 1086 Clade C Elicits Durable Humoral and Cellular Responses in Rhesus Macaques
Source: Mol Ther. 2016 Jul 26;24(11):2021–32. doi: 10.1038/mt.2016.123 (PMC5154473; doi:10.1038/mt.2016.123)
Supplement: Supplementary Figure S1 [file mt2016123x1.pdf]

Figure S1

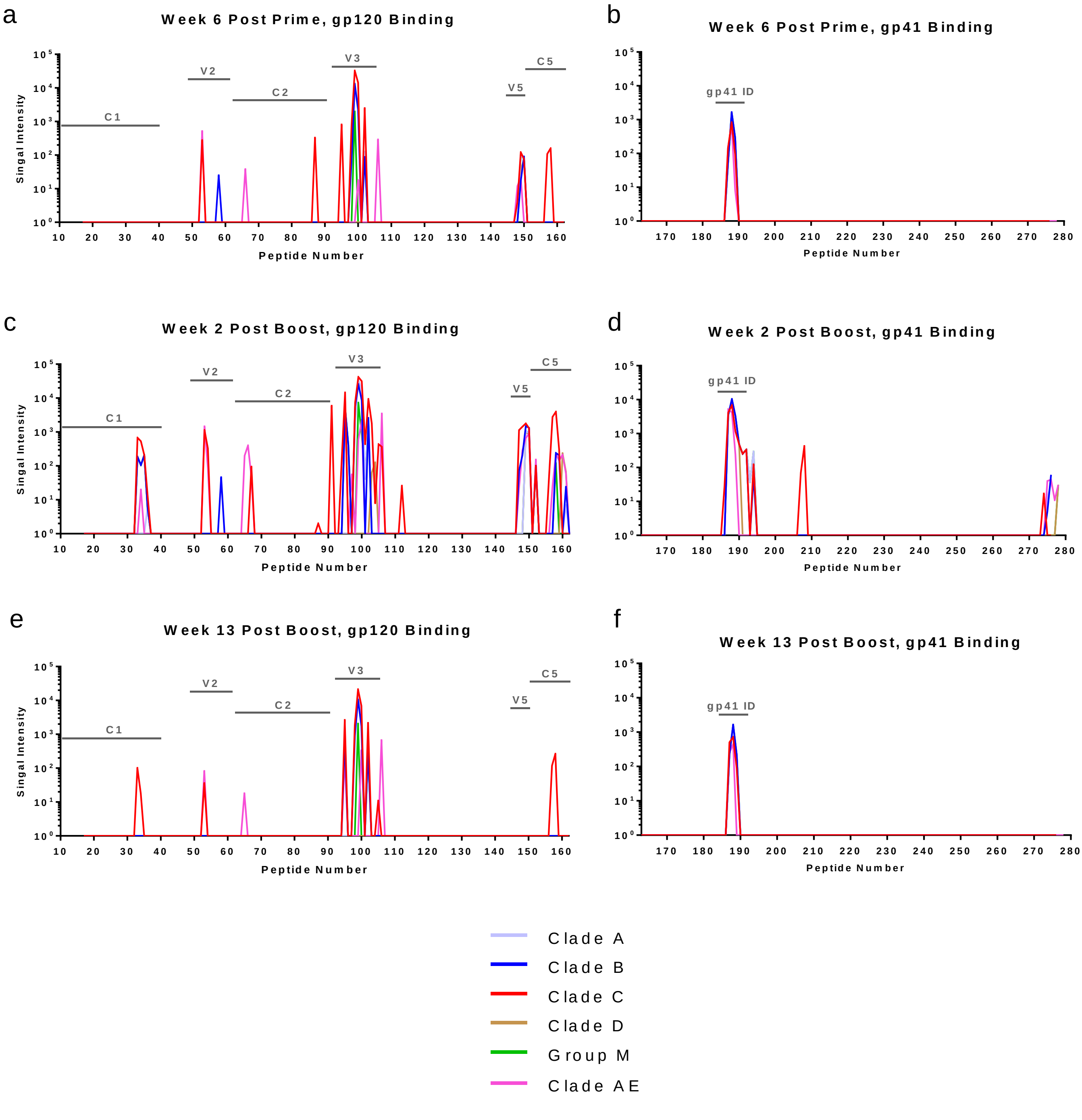

Figure S1. Median binding intensity of the 6 animals immunized animals against cross-clade gp120 (left) and gp41 (right) sequences, at 2 weeks post prime **(a-b)**, 6 weeks **(c-d)** and 13 weeks **(e-f)** post boost.
